# Targeting of C-ROS-1 Activity Using a Controlled Release Carrier to Treat Craniosynostosis in a Preclinical Model of Saethre-Chotzen Syndrome

**DOI:** 10.1155/2024/8863925

**Published:** 2024-05-09

**Authors:** Esther Camp, Laura Gonzalez Garcia, Clara Pribadi, Sharon Paton, Krasimir Vasilev, Peter Anderson, Stan Gronthos

**Affiliations:** ^1^Mesenchymal Stem Cell Laboratory, School of Biomedicine, Faculty of Health and Medical Sciences, The University of Adelaide, Adelaide, SA, Australia; ^2^Precision Medicine Theme, South Australian Health and Medical Research Institute, Adelaide, SA, Australia; ^3^School of Engineering and Future Industries Institute, Mawson Lakes Campus, University of South Australia, Mawson Lakes, SA, Australia; ^4^College of Medicine and Public Health, Flinders University, Bedford Park, SA, Australia; ^5^Cleft & Craniofacial SA, Woman's and Children's Hospital, Adelaide, SA, Australia; ^6^Adelaide Medical School, The University of Adelaide, Adelaide, SA, Australia

## Abstract

Saethre-Chotzen syndrome (SCS) is one of the most prevalent craniosynostosis, caused by a loss-of-function mutation in the *TWIST-1* gene, with current treatment options relying on major invasive transcranial surgery. *TWIST-1* haploinsufficient osteogenic progenitor cells exhibit increased osteogenic differentiation potential due to an upregulation of the transmembrane tyrosine kinase receptor, *C-ROS-1*, a TWIST-1 target gene known to promote bone formation. The present study assessed the efficacy of suppressing C-ROS-1 activity using a known chemical inhibitor to C-ROS-1, crizotinib, to halt premature coronal suture fusion in a preclinical mouse model of SCS. Crizotinib (1 *μ*M, 2 *μ*M, or 4 *μ*M) was administered locally over the calvaria of Twist‐1^del/+^ heterozygous mice prior to coronal suture fusion using either a nonresorbable collagen sponge (quick drug release) or a resorbable sodium carboxymethylcellulose microdisk (slow sustained release). Coronal suture fusion rates and bone parameters were determined by *μ*CT imaging and histomorphometric analysis of calvaria postcoronal suture fusion. Results demonstrated a dose-dependent increase in the efficacy of crizotinib to maintain coronal suture patency, with no adverse effects to brain, kidney, liver, and spleen tissue, or blood cell parameters. Moreover, crizotinib delivered on microdisks resulted in a greater efficacy at a lower concentration to reduce bone formation at the coronal suture sites compared to sponges. However, the bone inhibitory effects were found to be diminished by over time following cessation of treatment. Our findings lay the foundation for the development of a pharmacological nonsurgical, targeted approach to temporarily maintain open coronal sutures in SCS patients. This study could potentially be used to develop similar therapeutic strategies to treat different syndromic craniosynostosis conditions caused by known genetic mutations.

## 1. Introduction

Craniosynostosis is a medical condition involving partial or complete premature fusion of the cranial sutures, which bridge the osteogenic fronts of the cranial bone plates [1]. Fusion of the cranial plates occurs postnatally, as sutures normally remain patent in young children, allowing for the expansion of the skull to accommodate the growing brain [2, 3]. Premature fusion of the cranial sutures can result in increased intracranial pressure that can result in optic atrophy, blindness, and developmental delay [4]. Moreover, children with just unilateral or bilateral suture craniosynostosis are prone to developing cognitive, language, and motor skill difficulties during infancy and childhood [5]. Craniosynostosis can occur either as an isolated event, resulting in nonsyndromic craniosynostosis due to unknown mechanisms, or as a result of specific genetic mutations in clinically recognised syndromes. One of the most prevalent syndromic craniosynostosis, Saethre-Chotzen syndrome (SCS), occurs at an incidence of up to 1 in 25,000 births, primarily involving unilateral or bilateral coronal synostosis and mild limb deformities such as brachydactyly and cutaneous syndactyly [6]; P. J. [7]. The underlining cause of SCS is through the inheritance of an autosomal-dominant loss-of-function mutation in the *TWIST-1* gene, a basic helix-loop-helix transcription factor involved in skeletal and craniofacial development [8]. To date, there are over one hundred different heterozygous mutations identified in the *TWIST-1* gene, all of which result in *TWIST-1* haploinsufficiency and varying phenotypes of SCS, ranging from isolated unilateral coronal craniosynostosis to the extreme manifestation of multiple suture involvement [8–10]; D. [11]. At present, the only available treatment option is invasive transcranial surgery to increase skull volume and to correct for any asymmetry of the face and skull. Furthermore, multiple surgical interventions are often required in infancy, which can be associated with postoperative complications including neurological damage, blindness, infection, site morbidity, and even death. The development of new nonsurgical approaches with therapeutic agents to prevent or minimize fusion could have a profound impact on the treatment and management of SCS. Recent studies have assessed a variety of drug delivery systems to potentially treat prefusion of cranial sutures, such as the use of the BMP signalling inhibitors, glypicans, and Noggin, delivered on either titania nanotube arrays or PGLA microspheres to reduce cranial bone cell differentiation in vitro and in vivo [12, 13]; W. [14]. Conversely, PEG-based hydrogel drug delivery systems have also been used to deliver BMP-2 in order to promote cranial bone regeneration [15].

The tyrosine kinase receptor c-ros-oncogene 1 (C-ROS-1) has recently been identified as a novel TWIST-1 target [16]. C-ROS-1 belongs to the sevenless subfamily of tyrosine kinase insulin receptors and is conserved among vertebrates [17]. The tyrosine kinase receptor comprises a glycoprotein-rich extracellular domain, a transmembrane domain, and an intracellular tyrosine kinase (PT kinase) domain [18]. *C-ROS-1* is expressed postnatally in adult lung, kidney, heart, intestine, and epididymis [19, 20], and in osteogenic progenitors derived from calvaria and bone marrow [16, 21]. TWIST-1 regulates osteogenesis in part by silencing *C-ROS-1* expression, where C-ROS-1 activity promotes osteogenic differentiation in osteoprogenitor cells [16]. Importantly, the inhibition of C-ROS-1 activity was able to suppress bone formation in ex vivo expanded calvarial cells and whole calvarial explants derived from Twist‐1^del/+^ heterozygous mice, which exhibit craniofacial and limb abnormalities, similar to that described in SCS patients, including premature fusion of the coronal suture [22, 23] [24, 25]. The present study assessed the local administration of a C-ROS-1 chemical inhibitor, Crizotinib, delivered either on a nonresorbable collagen sponge or on resorbable sodium carboxymethylcellulose (CMC) microdisks, as a nonsurgical approach to treat craniosynostosis in Twist‐1^del/+^ mutant mice.

## 2. Materials and Methods

### 2.1. Microdisk Formulation

The microdisk carriers were prepared by solvent casting. Sodium carboxymethylcellulose (CMC, 90.000 *M*_*W*_, Sigma-Aldrich) was dissolved at 40 mg/mL in distilled water. Crizotinib stock solution of 50 mM in DMSO was used to prepare the dissolvable microdisks. Formulations containing increasing amounts of crizotinib to 25 *μ*M, 50 *μ*M, and 100 *μ*M were prepared by mixing 990 *μ*L of CMC with crizotinib solution to form 25 *μ*M, 50 *μ*M, and 100 *μ*M crizotinib solutions. The microdisks (approximately 4 mm diameter) were prepared by drying 20 *μ*L of the formulated solutions over a Teflon sheet at 40°C for 1 hour.

### 2.2. In Vitro Release Profile

The in vitro release profile of the formulated microdisks was carried out in a scalp-bone synthetic environment. Synthetic scalp was modelled by 25% w/v gelatin hydrogels which were prepared by dissolving 2.5 g of gelatin (Sigma-Aldrich) in 10 mL Milli-Q water at 40°C. Collagen-rich gelatin hydrogels were selected in this approach for the chemical structure, mechanical properties, and water content similarities with the subcutaneous environment [26–28]. Gelatin solution was then carefully pipetted into 12 well plates and cooled down to solidify forming 1 mm thick gels. Blank microdisks containing 25.22 nM Brilliant Blue G (Sigma-Aldrich) as drug surrogate were used to assess the release profile. The microdisks were placed on the surface of the plate under the gelatine layer, and a clear polycarbonate thin film was placed over the gelatin hydrogel to prevent evaporation. A BioTek EL800 Microplate reader was used to measure the absorbance at 630 nm in a matrix of 5 by 5 measurements per well separated by 2 mm centre to centre in an area of 1 cm^2^. Area absorbance measurements were taken at extended time periods of 2.5, 5, 7.5, 10, 15, 20, 25, 30, 35, 40, 45, and 60 min continued by hourly measurements until 24 hours. The drug diffusion area was measured, and the percentage of dose remaining in the centre of the microdisks was estimated by the decrease in the absorbance along time as given by the following equation:

(1) $$\begin{array}{c} \%\text{ dose remaining in centre}=\frac{A_{t}}{A_{t0}}\cdot 100. \end{array}$$

### 2.3. Drug Delivery

To assess the efficacy of crizotinib to halt precoronal suture fusion in Twist‐1^del/+^ heterozygous mice, crizotinib was delivered locally under the skullcap in Twist‐1^del/+^ mice at postnatal day 8 (P8). Crizotinib at 1 *μ*M, 2 *μ*M, and 4 *μ*M was administered over the calvaria of Twist‐1^del/+^ heterozygous mice prefusion of the coronal sutures (P8) using two delivery methods based on a concentration range previously used to assess osteogenesis in mouse calavrial cell cultures and whole calvarial explants in vitro [21]. Method 1 utilised a nonresorbable collagen sponge (quick drug release). Two 3 mm^3^ CollaCote sponges (Cat# 0101, Integra Life Sciences Services, Saint Priest, FRA) soaked in 0.1% DMSO as vehicle control or in crizotinib at 1 *μ*M, 2 *μ*M, and 4 *μ*M were placed subcutaneously onto each side of the coronal sutures of 8-day-old (P8) Twist‐1^del/+^ mice (Supplementary Figure 1). Method 2 utilised resorbable sodium carboxymethylcellulose (CMC) microdisks (4 mm) containing either 1 *μ*M, 2 *μ*M, or 4 *μ*M crizotinib overlayed at the midline over the top of the two coronal sutures of P8 Twist‐1^del/+^ mice (Supplementary Figure 1). Coronal suture fusion rates and bone parameters were analysed at postnatal day 20 (P20) and postnatal day 25 (P25) at postcoronal suture fusion. Animal experiments were performed in accordance with SAHMRI Animal Ethics Committee Approval (protocol number: SAM262).

### 2.4. Microcomputed Tomography

P20 and P25 mouse skulls were evaluated using microcomputed tomography (*μ*CT; SkyScan 1076 X-ray Micro-CT; Bruker microCT, Kontich, Belgium) at 9 mm resolution, AI 0.5 mm filter, excitation 990 ms, voltage 50 kV, current 500 *μ*A, rotation step 0.4, and 3-frame averaging. Reconstruction of the original scan data was performed using NRecon reconstruction (64 bit, ver. 1.6.10; Bruker microCT) with a smoothing of 1, ring artifact of 10, and beam hardening of 30%. Reconstructed.bmp files were realigned in data viewer (64 bit, ver.1.5.2; Bruker microCT) using Dragonfly 2020.2 Computer software (Object Research Systems (ORS) Inc, Montreal, Canada, 2020; https://www.theobjects.com/dragonfly).

### 2.5. Tissue Histology and Histomorphometric Analysis

Calvaria from P20 and P25 Twist‐1^del/+^ mice treated with control or various concentrations of crizotinib at P8 using either CollaCote sponges or CMC microdisks were fixed in 10% formalin overnight and decalcified for two days (14% EDTA, pH 7.2). Brain, kidney, liver, and spleen tissues were isolated from P25 Twist‐1^del/+^^*+*^ mice treated with control or various concentrations of crizotinib at P8, using CMC, and were isolated and fixed in 10% formalin overnight. All tissues were dehydrated and embedded in paraffin. Transverse sections (7 *μ*m) were cut and deparaffinized in xylene, rehydrated in decreasing concentrations of ethanol and stained with Masson's trichrome. Sections from calvaria were used to perform histomorphometric analysis. The formation of mineralized bone shown in blue was measured and histomorphometric analysis of mineralized bone formed and bone density (bone volume/total volume) were determined using OsteoMeasure XP Advanced Bone Histomorphometry ver.1.0.3.1 software (OsteoMetrics, Inc., Decatur, GA, US) on an Olympus BX53Microscope (Olympus, Notting Hill, VIC, AUS). Apoptosis in situ was measured using the DeadEnd™ Colorimetric TUNEL Kit (Promega corp. Madison, WI) according to the manufacturer's instructions.

### 2.6. Blood Cell Analysis

Total blood was collected in 0.5M EDTA from P25 Twist‐1^del/+^ mice treated with control or various concentrations of crizotinib in CMC microdisks via cardiac puncture. Blood samples were analysed using HEMAVET HV950FS system (Drew Scientific, Miami Lakes, FL).

## 3. Statistics

Experiments were performed in triplicates. Data analysis was carried out using Microsoft GraphPad Prism 5 (GraphPad Software, RRID: CR_002798, ), which was used for the generation of graphs and calculation of statistical significance using either the unpaired *T*-test for two variable comparisons or one-way ANOVA with the Tukey post hoc multiple comparison test, *p* < 0.05.

## 4. Results

### 4.1. CMC as a Potential Carrier Vehicle for Local Delivery of Crizotinib to Treat Craniosynostosis

Subcutaneous microdisks are becoming a popular form of parenteral sustained release dosage as they avoid the need for more invasive surgeries. Biodegradable polymers are preferred for the manufacture of these devices as they can be metabolised and excreted by the body, therefore avoiding the need for surgical removal at the end of the treatment [29]. One of these commonly used formulation materials is carboxymethylcellulose, a natural linear polysaccharide with excellent biocompatibility and biodegradability widely used in drug delivery processes [30] approved by the FDA for human pharmacological use (“Code of federal regulations”: FDA department of health and human services, Title 21, Volume 5, Chapter 1, Subchapter D, Drugs for human use (21CFR310.545),” 2023). A diversity of processes guide the drug release kinetics of sustained formulations ranging from the wetting and swelling to the erosion or degradation of the delivery vehicle, followed by the drug diffusion and permeation through the subcutaneous tissue.

**Figure 1:**
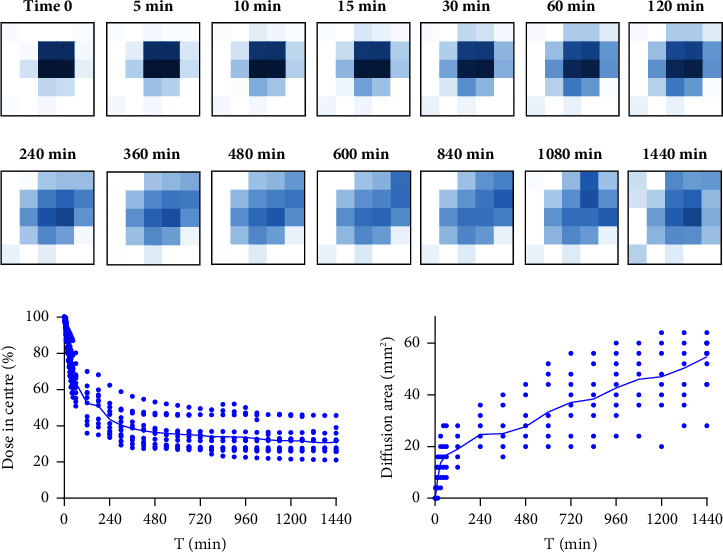
In vitro dissolution profile of the formulated CMC microdisks. (a) Heatmap diagram of the microdisks dissolution. Diagrams show the absorbance intensity measured in an area of 1 cm^2^. The Brilliant Blue G drug surrogate model contained in the microdisks releases at the synthetic scalp and diffuses between the skin-bone space along time. (b) Brilliant blue G release profile of the formulated microdisks. (c) Increment of the microdisks diffusion area along time. Data represented as the mean ± SD (*n* = 5–12 replicates per time point).

After subdermal application, the CMC microdisks come into contact with the subcutaneous tissue and become exposed to the interstitial fluid. It is in this environment that the microdisks start releasing crizotinib following three different stages. First, the moisture from the subdermal environment permeates through the microdisks. Then, the swelled matrix starts releasing the drug into the surrounding tissue; and third, the free drug is transported through the hydrated discs into the subdermal surroundings [31]. Currently, there is no regulatory guidance for the in vitro release testing of extended-release parenteral products, and expert working groups have identified the need for these standards [32–34]. Conventional in vitro USP tests evaluate the drug release from a tablet or capsule in a vessel containing large volumes of buffer (∼1L) at 37°C. However, this approach designed for oral dosing does not simulate the drug diffusion and mass transport phenomena of the in vivo subcutaneous administration. Hydrogel-based in vitro studies are used as a better model to simulate the diffusion and transport mimicking the subcutaneous tissue [35]. In this work, a collagen-rich hydrogel was used to characterize the release rate of the CMC microdisks. We used a model compound Brilliant Blue G as a drug surrogate to allow us to visualise the release mechanisms.  Figure 1(a) shows a graphical representation of the in vitro release profile of the studied CMC microdisks. The absorbance intensity was measured in a 1 cm^2^ area at set time points over 24 hours. A decrease of the absorbance intensity (lighter blue colours) is observed after the 1-hour time point. This is due to a fast swelling of the microdisks caused by the initial water absorption into the solid matrix followed by a release of the drug surrogate (Brilliant Blue G) from the depot formulation.  Figure 1(b) plots the percentage of Brilliant Blue G drug remaining at centre of the microdisks. Similarly, a rapid increase in Brilliant Blue G diffusion occurred in the first hour of in vitro contact followed by a slow release that tends to the asymptote after 8 hours. The dose remaining in the centre of the microdisks was 75.4% after 30 minutes, indicating the swelling phenomena. The subsequent release is a slower process with 63.9% of dose remaining in the centre at 1 hour, 50.1% of dose at 3 hours, 40.4% at 5 hours, and 36,4% at 8 hours. The final steps of the curve were marked by Brilliant Blue G diffusion into the gel, with 33.6% of dose remaining at 16 hours and 30.9% at 24 hours. It is worth noting that this approach is a closed system with no sink and no flow; therefore, the dose remaining when the release is complete will reach an equilibrium concentration in the well. The microdisk diffusion area was measured on the spectrophotometric areas along time. The fast-swelling effect was observed in the first 30 minutes, after which the diffusion area of Brilliant Blue G into the hydrogel continued to increase at a slower consistent rate for 24 hours (Figure 1(c)). It should be noted that the molecular weight of Brilliant Blue G is lower than that of crizobinib which is likely to result in a slower release rate. However, the data presented in Figure 1 are useful to visualise the mechanism of release from the microdisks.

### 4.2. Local Administration of Crizotinib in CMC Microdisks Reduces Calvarial Suture Closure in Twist-1^del/+^ Mutant Mice

**Figure 2:**
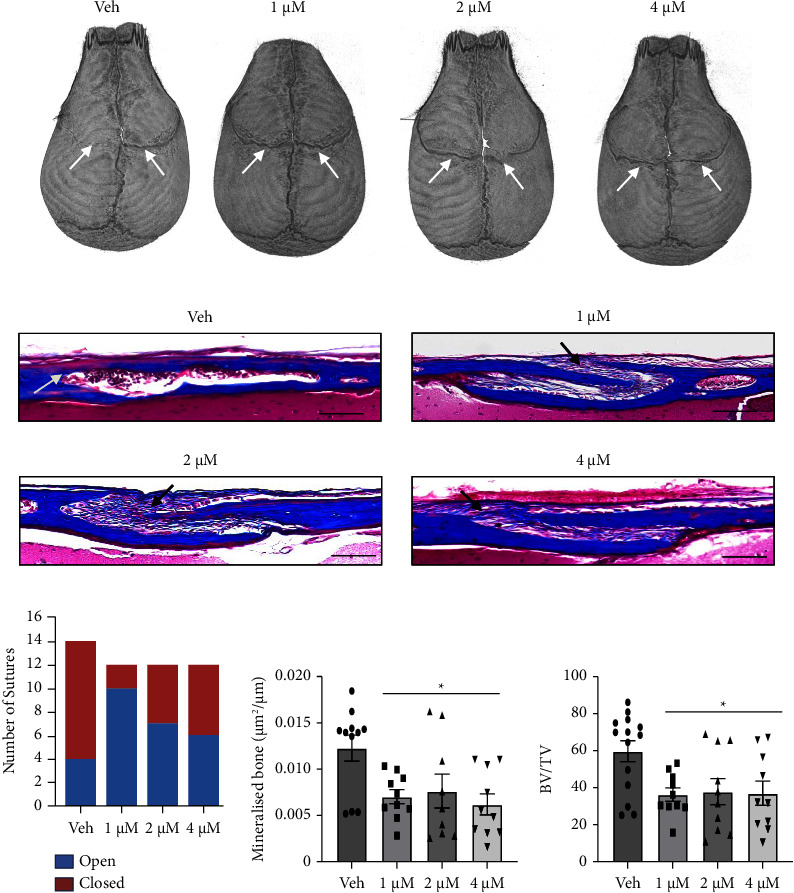
Local administration of CMC microdisk infused with crizotinib reduces calvarial bone formation and the incidence of premature coronal suture fusion in 20 -day-old Twist‐1^del/+^ mice. (a) Representative *μ*CT images of coronal sutures (arrows) of 20-day-old Twist‐1^del/+^ mouse skulls following local implantation of CMC microdisks containing either 0.1% DMSO or crizotinib at different concentrations (1-4 *μ*M) at P8. (b) Representative images of Masson's trichome-stained coronal suture sections (white arrow: closed sutures, black arrow: open sutures) from 20-day-old Twist‐1^del/+^ mice following local implantation a CMC microdisc containing either 0.1% DMSO or crizotinib at different concentrations (1–4 *μ*M), scale bar = 100 *μ*m (100× magnification). (c) Number of open coronal sutures versus closed coronal sutures in crizotinib-treated Twist‐1^del/+^ mice at P20 (*n* = 12 per the treatment group). (d) Histomorphometric analysis of mineralized bone formed of locally treated coronal sutures. Values are the mean ± SEM, ^*∗*^*p* < 0.05 one-way ANOVA with Tukey's multiple comparisons, DF = 3, *F* = 4.567 (1 *μ*M Crizo, *p*=0.018; 2 *μ*M Crizo, *p*=0.048; 4 *μ*M Crizo, *p*=0.005), *n* = 8–11 Twist‐1^del/+^ coronal sutures/treatment group. (e) Histomorphometric analysis of bone volume fraction (bone volume/total volume) of locally treated coronal sutures. Values are the mean ± SEM, ^*∗*^*p* < 0.05, one-way ANOVA with Tukey's multiple comparisons, DF = 3, *F* = 1.637 (1 *μ*M Crizo, *p*=0.017; 2 *μ*M Crizo, *p*=0.029; 4 *μ*M Crizo, *p*=0.022), *n* = 9–14 Twist‐1^del/+^ coronal sutures/treatment group.

The present study examined the efficacy of a pharmacological approach to suppress craniosynostosis through the inhibition of C-ROS-1 activity with crizotinib in Twist‐1^del/+^ mutant mice as a potential novel target for the treatment of SCS. Previous studies have described the suppression of osteogenesis in cultures of mouse calvaria bone cells and in calvarial explant cultures, in the presence of the C-ROS-1 inhibitor, crizotinib [21]. The present study assessed the efficacy of crizotinib to halt premature coronal suture fusion in Twist‐1^del/+^ heterozygous mice, where coronal suture closure begins at postnatal day 9. Controlled release resorbable CMC microdisks were seeded with crizotinib at 1 *μ*M, 2 *μ*M, and 4 *μ*M or vehicle alone (DMSO) and then implanted under the skull cap over the corona sutures of Twist‐1^del/+^ mice at P8 (precoronal fusion, Supplementary Figure 2) of age precoronal suture fusion. Following treatment with Crizotinib, the coronal sutures of P20 (postcoronal fusion) old Twist‐1^del/+^ mice were found to be opened in comparison to vehicle controls as assessed by µCT imaging (Figure 2(a)). Representative images of Masson's trichome-stained coronal suture sections of calvaria at P20 of treated Twist‐1^del/+^ mice confirmed the presence of open sutures at all the crizotinib concentrations tested, compared to the DMSO vehicle control, which showed closed coronal sutures (Figure 2(b)). Interestingly, the number of open coronal sutures versus closed coronal sutures appeared to decrease at the higher crizotinib concentrations (Figure 2(c)), which may be a consequence of local cell toxicity. Histomorphometric analysis of mineralized bone formed and bone density (bone volume/total volume) of locally treated coronal sutures showed significantly reduced levels across all the crizotinib concentrations compared to the vehicle control but not between the treatment groups (Figure 2(d), 2(e)).

**Figure 3:**
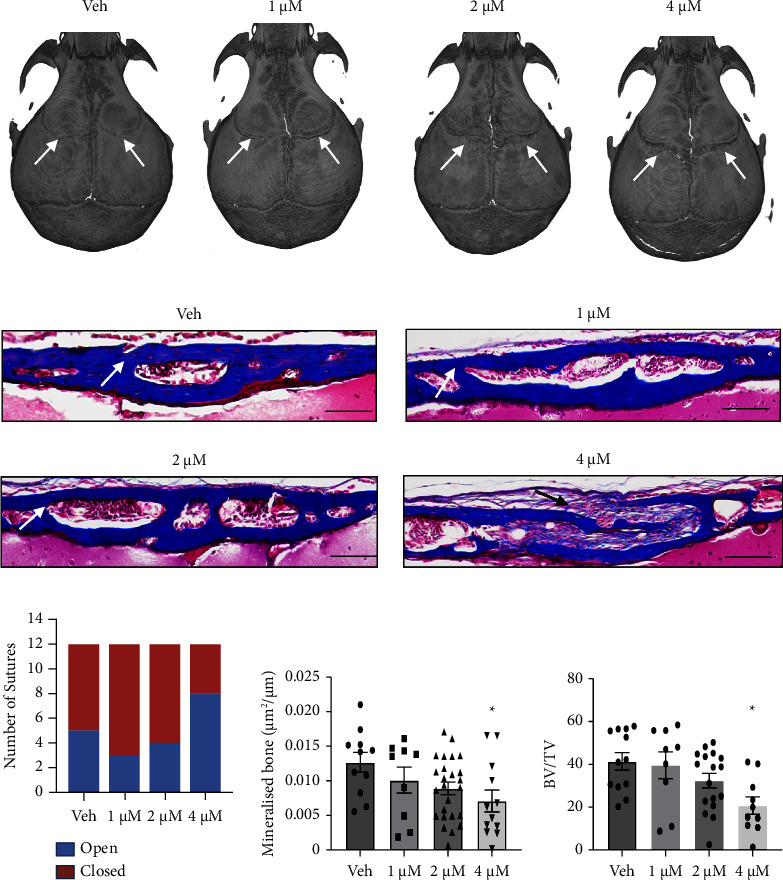
Local administration of CollaCote fragments infused with crizotinib reduces coronal suture fusion in 20-day-old Twist‐1^del/+^ mice. (a) Representative *μ*CT images of coronal sutures (arrows) of 20-day-old Twist‐1^del/+^ mouse skulls following local implantation of CollaCote sponge carriers containing either 0.1% DMSO or crizotinib at different concentrations (1-4 *μ*M) at P8. (b) Representative images of Masson's trichome-stained coronal sutures (white arrow: closed sutures, black arrow: open sutures) of 20-day-old Twist‐1^del/+^ mice following local implantation of CollaCote sponge carriers containing either 0.1% DMSO or 4 *μ*M crizotinib at P8, scale bar = 100 *μ*m (100×). (c) Number of open coronal sutures versus closed coronal sutures in crizotinib-treated Twist‐1^del/+^ mice at P20 (*n* = 12 per the treatment group). (d) Histomorphometric analysis of mineralized bone formed of locally treated coronal sutures. Values are the mean ± SEM, ^*∗*^*p* < 0.05, one-way ANOVA with Tukey's multiple comparisons, DF = 3, *F* = 2.623 (1 *μ*M Crizo, *p*=0.655; 2 *μ*M Crizo, *p*=0.165; 4 *μ*M Crizo, *p*=0.043), *n* = 9–21 Twist‐1^del/+^ coronal sutures/treatment group. (e) Histomorphometric analysis of bone volume fraction (bone volume/total volume) of locally treated coronal sutures. Values are the mean ± SEM, ^*∗*^*p* < 0.05, one-way ANOVA with Tukey's multiple comparisons, DF = 3, *F* = 4.193 (1 *μ*M Crizo, *p*=0.986; 2 *μ*M Crizo, *p*=0.261; 4 *μ*M Crizo, *p*=0.005), *n* = 9–17 Twist‐1^del/+^ coronal sutures/treatment group.

Parallel studies were performed to compare the potency of crizotinib treatment using a nonresorbable CollaCote collagen sponge delivery system (rapid uncontrolled drug release) implanted under the skullcap in Twist‐1^del/+^ mice at P8. Coronal sutures of P20 old Twist‐1^del/+^ mice were observed to be patent predominantly in those mice treated with the highest dose of crizotinib (4 *μ*M) compared to either the DMSO control or lower doses of crizotinib based on µCT imaging (Figure 3(a)). Representative images of Masson's trichome-stained coronal suture sections of P20 Twist‐1^del/+^ confirmed that the majority of open sutures were present in mice treated with 4 *μ*M crizotinib compared to the DMSO control and lower doses of crizotinib (Figures 3(b) and 3(c)). Moreover, histomorphometric analysis showed significantly reduced levels of mineralized bone formation and bone density in mice that received the 4 *μ*M crizotinib delivered with CollaCote sponge when compared to the vehicle control but not between other treatment groups (Figure 3(d), 3(e)). Overall, these studies indicate that the suppression of craniosynostosis occurs in the presence of a one-off, locally delivered crizotinib, where efficacy is dependent on the delivery approach used, favouring a resorbable polymer system with controlled drug release properties.

### 4.3. The Effect of Local Administration of Crizotinib on Calvarial Suture Closure in Twist-1^del/+^ Mice was found to be Reversible with No Signs of Any Side Effects

**Figure 4:**
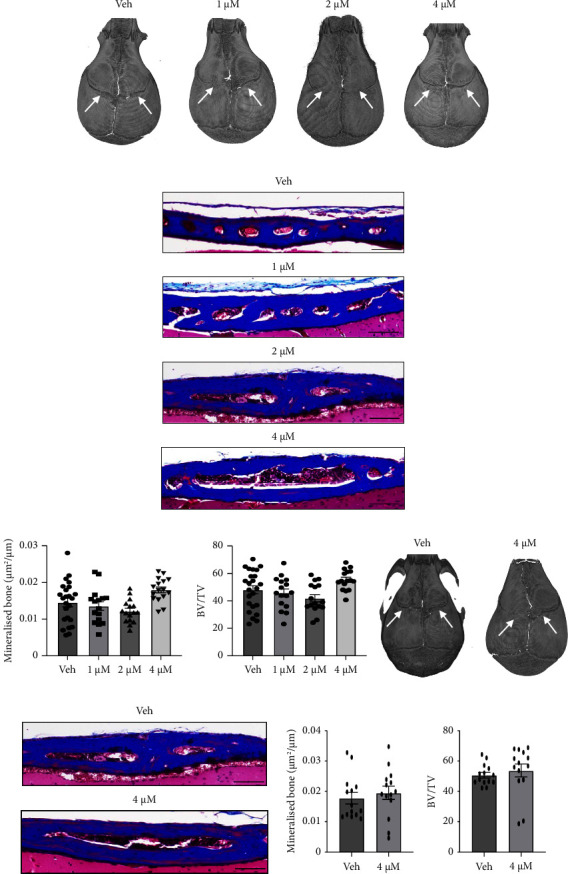
Crizotinib treatment is reversible in Twist‐1^del/+^ mice by postnatal day 25. (a) Representative *μ*CT images of coronal sutures (arrows) of 25-day-old Twist‐1^del/+^ mouse skulls following local implantation of CMC microdiscs containing either 0.1% DMSO or crizotinib at different concentrations (1–4 *μ*M) at P8. (b) Representative images of Masson's trichome-stained coronal sutures sections from 25-day-old Twist‐1^del/+^ mice following local implantation of CMC microdisc containing either 0.1% DMSO or 1, 2, or 4 *μ*M of crizotinib at P8, scale bar = 100 *μ*m (100×). (c) Histomorphometric analysis of mineralized bone formed of locally treated coronal sutures. Values are the mean ± SEM, one-way ANOVA with Tukey's multiple comparisons, DF = 3, *F* = 2.63, (1 *μ*M Crizo, *p*=0.855; 2 *μ*M Crizo, *p*=0.268; 4 *μ*M Crizo, *p*=0.056), *n* = 16–26 Twist‐1^del/+^ coronal sutures/treatment group. (d) Histomorphometric analysis of bone density (bone volume/total volume) of locally treated coronal sutures. Values are the mean ± SEM, one-way ANOVA with Tukey's multiple comparisons, DF = 3, *F* = 3.812 (1 *μ*M Crizo, *p*=0.892; 2 *μ*M Crizo, *p*=0.261; 4 *μ*M Crizo, *p*=0.139), *n* = 16–26 Twist‐1^del/+^ coronal sutures/treatment group. (e) Representative *μ*CT images of coronal sutures (arrows) for 25-day-old Twist‐1^del/+^ mouse skulls following local implantation of CollaCote sponge carriers containing either 0.1% DMSO or 4 *μ*M crizotinib at P8. (f) Representative images of Masson's trichome-stained coronal sutures sections from 25-day-old Twist‐1^del/+^ mice following local implantation of a CollaCote containing either 0.1% DMSO or 4 *μ*M crizotinib at P8, scale bar = 100 *μ*m (100×). (g) Histomorphometric analysis of mineralized bone formed of locally treated coronal sutures in mice treated with CollaCote containing either 0.1% DMSO or 4 *μ*M crizotinib. Values are the mean ± SEM, *p*=0.561, unpaired *t*-test, two-tailed (Crizo 4 *μ*M, *p*=0.561), *n* = 14 Twist‐1^del/+^ coronal sutures/treatment group. (h) Histomorphometric analysis of bone density (bone volume/total volume) of locally treated coronal sutures in mice treated with CollaCote containing either 0.1% DMSO or 4 *μ*M crizotinib. Values are the mean ± SEM, *p*=0.561, unpaired *t*-test, two-tailed (Crizo 4 *μ*M, *p*=0.523), *n* = 14 Twist‐1^del/+^ coronal sutures/treatment group.

Replicate animal cohorts were allowed to progress to the P25 stage to determine the longer lasting effects of drug treatment. Analysis of the effective treatment regimes, using either 1-4 *μ*M crizotinib seeded onto CMC (Figure 4(a)–4(d)) or 4 *μ*M crizotinib seeded onto CollaCote sponge (Figure 4(e)–4(h)) showed that coronal bone fusion had recommenced by P25. These data indicate that local delivery of crizotinib can regulate coronal fusion, where this process appears to be reversible allowing for the eventual fusion of sutures after a single treatment at the prefusion stage of P8.

**Figure 5:**
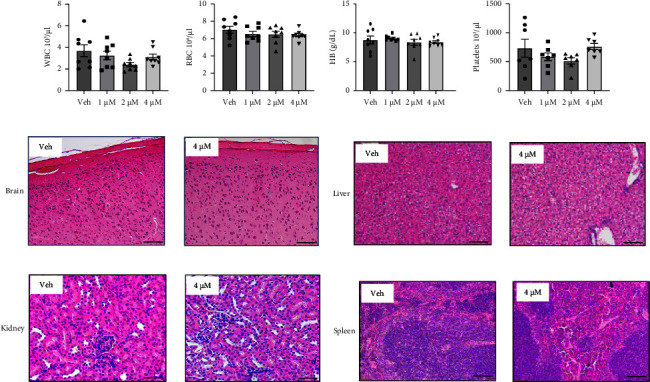
No significant adverse effects following CMC + crizotinib treatment. Measurement of blood parameters: (a) white blood cells (WBCs), (b) red blood cells (RBCs), (c) haemaglobin (HB), and (d) platelets were assessed for whole blood taken from 25-day-old Twist‐1^del/+^ mice following local implantation of CMC microdisks containing either 0.1% DMSO or 1–4 *μ*M crizotinib at P8. Values are the mean ± SEM, one-way ANOVA with Tukey's multiple comparisons, DF = 3, (a) *F* = 3.812 (1 *μ*M Crizo, *p*=0.751; 2 *μ*M Crizo, *p*=0.061; 4 *μ*M Crizo, *p* = 0.576), (b) F = 0.791 (1 *μ*M Crizo, *p*=0.698; 2 *μ*M Crizo, *p*=0.643; 4 *μ*M Crizo, *p*=0.502), (c) *F* = 0.557 (1 *μ*M Crizo, *p* = 0.932; 2 *μ*M Crizo, *p* = 0.855; 4 *μ*M Crizo, *p* = 0.894), (d) *F* = 1.652 (1 *μ*M Crizo, *p*=0.528; 2 *μ*M Crizo, *p*=0.241; 4 *μ*M Crizo, *p*=0.994), *n* = 7–8 Twist‐1^del/+^ mice/treatment group. Representative histological sections of H&E stained (e) brain tissue, (f) liver tissue, (g) kidney tissue, and (h) spleen tissue harvested from 25-day-old Twist‐1^del/+^ mice following local implantation of CMC microdisks containing either 0.1% DMSO or 1-4 *μ*M crizotinib at P8, scale bar = 50 *μ*m (100×).

The potential toxicity of drug therapy in Twist‐1^del/+^ mice at P25 was assessed following studies of the efficacy of 1-4 *μ*M crizotinib CMC treatment to transiently maintain coronal suture patency. Whole blood analysis found no statistical differences in the white cell counts, red cell counts, platelets counts, or haemaglobin levels between the different crizotinib concentrations and vehicle control group (Figure 5(a)–5(d)). Comparative histological assessment of the brain tissue underlying the coronal sutures showed no gross changes in tissue architecture or evidence of local inflammation at the highest crizotinib concentration tested (Figure 5(e)). Similarly, tissue sections of liver, kidney, and spleen appeared to exhibit normal tissue architecture with no signs of inflammation following treatment with 4 *μ*M crizotinib (Figure 5(f)–5(h)). Moreover, assessment of apoptosis in situ by TUNEL assay found no observable differences between vehicle controls and tissues treated with 4 *μ*M crizotinib (Supplementary Figure 3). These data indicate that a single application of crizotinib at prefusion has little adverse effects on local brain tissue and distal organ integrity.

## 5. Discussion

In SCS patients, the coronal sutures begin to fuse by 3 months of age, which normally fuse after 18 years of age in healthy individuals. The coronal sutures need to remain open for the first 2-3 years of postnatal development for the developing brain to reach a volume of 90%. In mice, the frontal sutures fuse by P15 [36], whilst all other sutures remain unfused throughout postnatal life [37]. However, in Twist‐1^del/+^ mice, the coronal sutures begin to fuse after P9 (equivalent to 2 months of age in humans) and are generally fully fused by P20 (approximately 3 years of age in humans). The present study assessed the efficacy and safety of crizotinib as a drug therapy to treat craniosynostosis in a preclinical model of SCS, using Twist‐1^del/+^ mutant mice. The rationale for this therapeutic strategy was based on previous studies, which found that the TWIST-1 transcription factor acts as a negative regulator of the *C-ROS-1* gene, where *TWIST-1* mutant SCS human cranial bone cells and Twist‐1^del/+^ mouse calvarial cells express higher levels of *C-ROS-1* [21]. Inhibition of either *C-ROS-1* gene expression or C-ROS-1 activity leads to a suppression of osteogenic potential in cultured human and mouse osteoprogenitor cells via deregulation of the PI3K/AKT signalling pathways [16, 21]. Moreover, treatment of Twist‐1^del/+^ mutant mouse calvaria bone explants with the C-ROS-1 chemical inhibitor, crizotinib, caused a decrease in mineral deposition compared to untreated calvaria explant cultures [21]. The small molecule tyrosine kinase inhibitor, crizotinib, targets C-ROS-1 [38], ALK [39], and C-MET [40] receptor tyrosine kinase activity and belongs to the 3-benzyloxy-2-aminopyridine series of kinase inhibitors [41]. Crizotinib acts by binding to the adenosine triphosphate (ATP)-binding site, blocking the binding of ATP and subsequent phosphorylation, resulting in suppressed enzymatic activity. The findings of the present study showed that coronal suture patency was maintained in a dose-dependent manner up to postnatal day 20 in Twist‐1^del/+^ mutant mice, following delivering of a single localized dose of *c*Crizotinib under the skull cap, at the precoronal suture fusion stage P8.

Comparative analysis of different scaffold delivery systems found that a control release CMC was more efficient at delaying coronal suture fusion with lower concentrations of Crizotinib, compared to a rapid release scaffold such as CollaCote sponge. CMC is a water-soluble polysaccharide with excellent biocompatibility, high hydrophilicity, and antifouling capability [42]. Moreover, CMC is biodegradable and undergoes enzymatic degradation, therefore, preventing bioaccumulation in subdermal tissues, properties which facilitate a slow sustained release at the implant site. For these reasons, this FDA-approved excipient is widely used on pharmaceutical formulations and wound healing applications (“Code of federal regulations: FDA department of health and human services, Title 21, Volume 5, Chapter 1, Subchapter D, Drugs for human use) [43]. The biocompatibility of the excipients used in subcutaneous implants is essential to avoid inflammatory responses that change the subcutaneous environment and affect drug absorption. In addition, the metabolic stability of the formulations in the subcutaneous tissue affects the bioavailability of the therapeutics. Nonetheless, the primary factor that influences drug absorption kinetics is the drug release and the mechanisms that govern it.

In vitro release into collagen-rich hydrogels is a biorelevant approach that mimics the complexity of the subcutaneous administration route. The UV-vis spectrophotometric continuous area readings allowed for the imaging observation of the kinetic release and drug diffusion processes in two dimensions. The data showed a fast release and drug diffusion during the first hour, suggesting a swelling effect of the subdermal microdisks after administration. After this process, the drug surrogate had a steady release for 8 hours and continued diffusing through the surrounding gel for 24 hours. This release assay can reflect the release mechanism of the subdermal microdisks but cannot be directly related to their in vivo performance. Further studies will consider the validation of this assay and determine the bioavailability of *c*Crizotinib to establish an in vitro-in vivo correlation. Overall, these properties make CMC an attractive drug delivery system for treating craniosynostosis with targeted inhibitors such as *c*Crizotinib. This is in contrast to using an uncontrolled release platform such as the commercially available nonresorbable, nontoxic collagen sponge, CollaCote, commonly used for wound dressing in dental surgery [44], and previously used as a drug delivery vehicle to treat craniosynostosis [45], but with a lower efficacy compared to CMC. Moreover, the application of *c*Crizotinib using CMC as a carrier to inhibit coronal suture prefusion in Twist‐1^del/+^ mutant mice was found to be comparable to previous studies assessing the delay of posterial frontal cranial suture fusion in wildtype mice, using PGLA microspheres to deliver the BMP signalling inhibitor, Noggin [14].

An important aspect of this study was the efficacy of targeting one biological factor known to be aberrantly expressed in SCS cranial bone cells, which can help alleviate a complex condition such as SCS. Importantly, the effect of a single dose of crizotinib on suture fusion was found to be reversible as the mice aged. The data showed that all treatment arms, which maintained coronal suture patency at P20, appeared to lose efficacy beyond this time point, leading to fusion of the majority of coronal sutures by P25 (equivalent to early adolescence stage of development in humans). The reversible nature of this approach implies that crizotinib therapy could be adjusted to temporally regulate coronal suture patency, depending on the severity of an individual SCS patient's condition.

Crizotinib (Xalkori, Pfizer, Inc.) was initially approved for the treatment of patients with metastatic ROS1-positive nonsmall cell lung cancer tumors, and has subsequently been approved for pediatric patients, 1 year of age and older with unresectable, recurrent, or refractory inflammatory ALK-positive myofibroblastic tumors [46]. Crizotinib treatment of patients is administered orally (generally 250 mg per day). While it has been generally reported to be well tolerated, crizotinib treatment has been associated with varying levels of toxicity in liver, kidney, gut, heart, and bone marrow [38, 47]; [48]. In the present study, a preliminary assessment was conducted of possible adverse effects in different tissues, following a single dose of crizotinib administered locally under the skull cap in developing mice. The data showed no adverse effects on blood cell parameters such as blood cell counts, platelet numbers, and haemaglobin levels. Furthermore, histological assessment on tissue integrity for brain, liver, kidney, and spleen showed no signs of inflammation or altered architecture in developing immature mice. However, further analysis remains to be performed in the future regarding any long-term effects in adult mice through more extensive assessment of tissue toxicity, skeletal growth, and behavioural studies, following Crizotinib treatment.

## 6. Conclusions

In summary, the present study has built on our previous discovery of a critical membrane bound tyrosine kinase molecule that is aberrantly expressed in SCS osteoprogenitor cells. This has led to the manipulation of endogenous osteogenic progenitors within the developing skull in a preclinical model of SCS that mimics the human condition. This study has led to the development of a nonsurgical approach to maintain the cranial sutures open in syndromic craniosynostosis caused by a known genetic mutation. Such a targeted approach may allow for normal brain development in the critical early years of postnatal development. These studies could have further implications for the development of novel pharmacological-based treatments targeting other deregulated target genes for different form of syndromic craniosynostosis.

## Data Availability

The data used to support the findings of this study are available from the corresponding author upon request.

## Supplementary Materials

Supplementary Materials Supplementary Figure 1: schematic representing surgical placement of (A) CMC disks and (B) CollaCote sponge over mouse cranium. Supplementary Figure 2: representative *μ*CT image of postnatal day 8 (P8) Twist‐1^del/+^ mutant mice cranium depicting open coronal sutures (arrows). Supplementary Figure 3: representative histological sections of TUNEL stained (A) brain tissue, (B) liver tissue, (C) kidney tissue, and (D) spleen tissue harvested from 25-day-old Twist‐1^del/+^ mice following local implantation of CMC microdisks containing either 0.1% DMSO (Veh) or 4 *μ*M crizotinib at P8 (200× magnification).
