# Supplementary figures and images for: Targeting of C-ROS-1 Activity Using a Controlled Release Carrier to Treat Craniosynostosis in a Preclinical Model of Saethre-Chotzen Syndrome
Source: J Tissue Eng Regen Med. 2024 May 9;2024:8863925. doi: 10.1155/2024/8863925 (PMC11919205; doi:10.1155/2024/8863925)

**A**

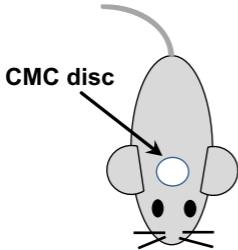

**B**

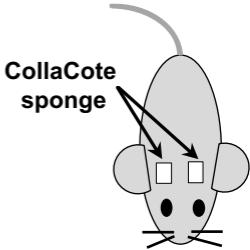

Supplement: Supplementary Materials — Supplementary Figure 1: schematic representing surgical placement of (A) CMC disks and (B) CollaCote sponge over mouse cranium. Supplementary Figure 2: representative μCT image of postnatal day 8 (P8) Twist‐1del/+ mutant mice cranium depicting open coronal sutures (arrows). Supplementary Figure 3: representative histological sections of TUNEL stained (A) brain tissue, (B) liver tissue, (C) kidney tissue, and (D) spleen tissue harvested from 25-day-old Twist‐1del/+ mice following local implantation of CMC microdisks containing either 0.1% DMSO (Veh) or 4 μM crizotinib at P8 (200× magnification). [file 8863925.f1.zip › Supplementary Figure 1.pdf]

***P8 Twist-1<sup>del/+</sup>* mouse cranium**

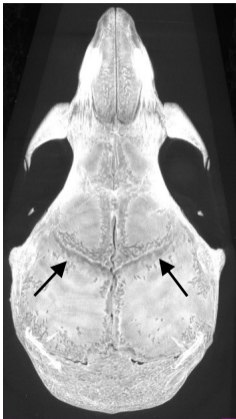

Supplement: Supplementary Materials — Supplementary Figure 1: schematic representing surgical placement of (A) CMC disks and (B) CollaCote sponge over mouse cranium. Supplementary Figure 2: representative μCT image of postnatal day 8 (P8) Twist‐1del/+ mutant mice cranium depicting open coronal sutures (arrows). Supplementary Figure 3: representative histological sections of TUNEL stained (A) brain tissue, (B) liver tissue, (C) kidney tissue, and (D) spleen tissue harvested from 25-day-old Twist‐1del/+ mice following local implantation of CMC microdisks containing either 0.1% DMSO (Veh) or 4 μM crizotinib at P8 (200× magnification). [file 8863925.f1.zip › Supplementary Figure 2.pdf]

**A**

Brain

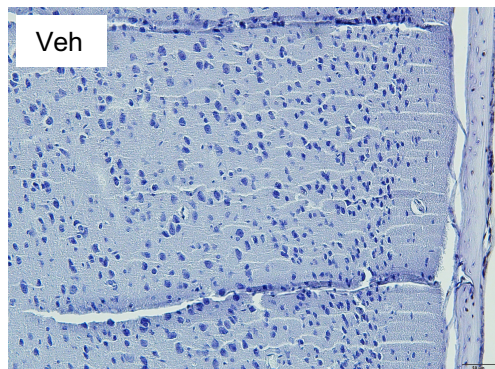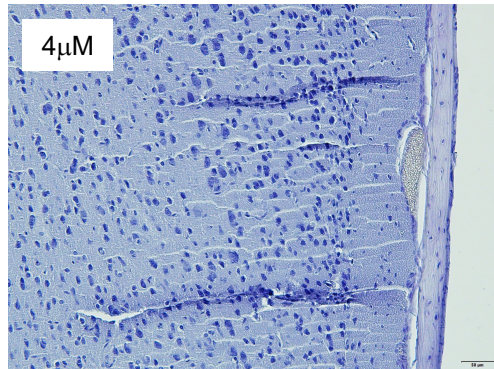**B**

Liver

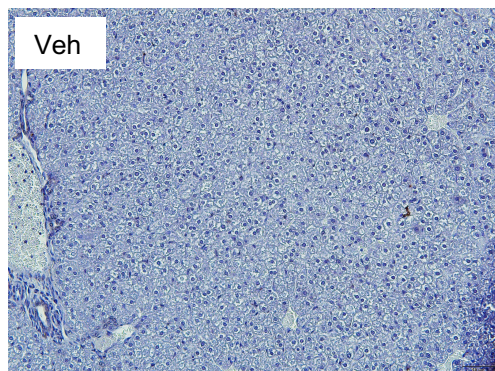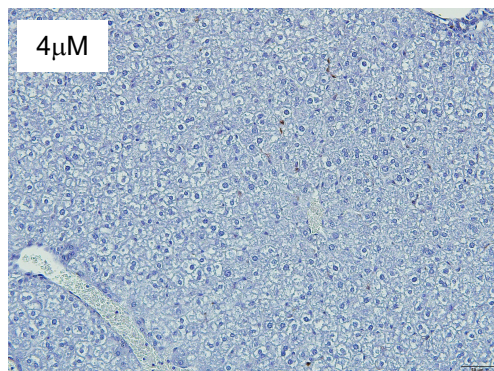**C**

Kidney

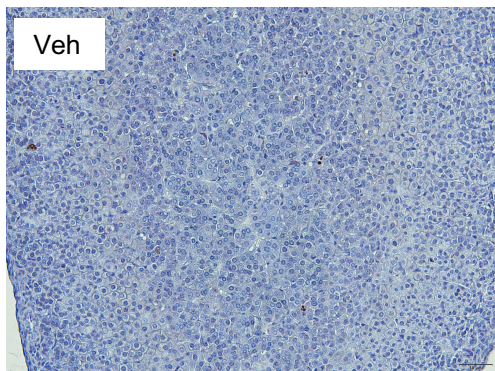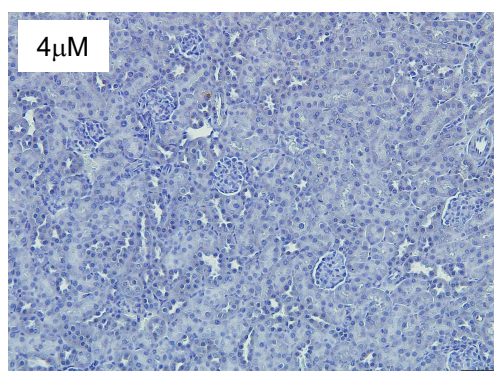**D**

Spleen

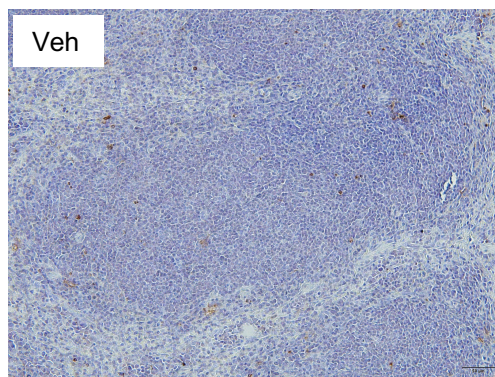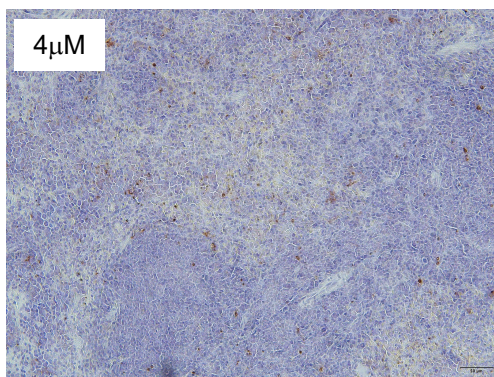

Supplement: Supplementary Materials — Supplementary Figure 1: schematic representing surgical placement of (A) CMC disks and (B) CollaCote sponge over mouse cranium. Supplementary Figure 2: representative μCT image of postnatal day 8 (P8) Twist‐1del/+ mutant mice cranium depicting open coronal sutures (arrows). Supplementary Figure 3: representative histological sections of TUNEL stained (A) brain tissue, (B) liver tissue, (C) kidney tissue, and (D) spleen tissue harvested from 25-day-old Twist‐1del/+ mice following local implantation of CMC microdisks containing either 0.1% DMSO (Veh) or 4 μM crizotinib at P8 (200× magnification). [file 8863925.f1.zip › Supplementary Figure 3.pdf]
